# Organizational Readiness for Change in the Era of Smart Hospital Wards: Qualitative Study of Health Care Workers’ Insights

**DOI:** 10.2196/81932

**Published:** 2025-12-18

**Authors:** Hui Wen Lim, Jennifer Sumner, Abigail Ang, Camille Keck, Emily Hwee Hoon Chew, Alexander Wenjun Yip

**Affiliations:** 1 Medical Affairs – Research Innovation & Enterprise Alexandra Hospital Singapore, Singapore Singapore; 2 Department of Healthcare Redesign Alexandra Research Centre for Healthcare in a Virtual Environment (ARCHIVE) Alexandra Hospital Singapore Singapore

**Keywords:** organizational readiness for change, health care innovation, technology adoption, change management, qualitative research

## Abstract

**Background:**

Technology is rapidly reshaping conventional hospital environments into smart spaces, enhancing care, improving clinical workflows, and reducing workloads. However, successful implementation depends not only on the effectiveness of the technology but also on organizational readiness for change.

**Objective:**

This study aimed to identify the key enablers and barriers to readiness for change for a smart hospital ward initiative.

**Methods:**

We conducted a qualitative study to gauge organizational readiness for change for a smart ward initiative. Using purposive sampling, we captured diverse views from clinicians, IT staff, operational support staff, and health care redesign staff. Data were coded deductively under 3 key domains in Weiner’s theory of organizational readiness: change efficacy, change commitment, and contextual factors. Subthemes were derived inductively under each domain.

**Results:**

We interviewed 19 participants, including clinicians and support staff. Six subthemes emerged: (1) perceived valence and feasibility; (2) transparency and trust in management; (3) shared understanding and readiness to act; (4) resources, training, and staff capability; (5) innovation culture; and (6) past experiences. Participants viewed the initiative as valuable and were motivated to change, citing that the institution’s innovation culture was a key enabler. However, there were key barriers, including unclear timelines, inconsistent training, limited resources, and a lack of infrastructure to support innovation. Concerns about overreliance on technology were also prominent, with staff wary of its impact on clinical judgment and system reliability.

**Conclusions:**

Enabling readiness for the smart ward initiative requires transparent communication of timelines and project awareness, particularly for ground staff, the development of training frameworks, and adequate prioritization of innovation. Alleviating commonly reported technology concerns, such as overreliance, loss of human touch, and system reliability, will also be key to adoption and sustainability.

## Introduction

Technology is rapidly transforming inpatient care, reshaping conventional hospital environments into smart spaces, including wards [1,2]. “Smart wards,” an emerging feature of digitally enabled hospitals, are ecosystems that use interconnected digital systems to enhance patient care, improve clinical workflows, and reduce workload through automation [3,4]. These ecosystems may incorporate a range of technologies, including real-time patient monitoring, movement detection, and artificial intelligence (AI)–based support tools [4]. Motivated by the challenges of caring for an aging population, a constrained health care workforce, and limited hospital bed capacity [5-7], there is growing investment in smart ward environments. However, the implementation of such technologies requires more than hardware or software; it demands meaningful behavioral and workflow changes among staff.

Alexandra Hospital is the latest hospital to invest in a smart ward concept as part of its commitment to adopting transformative health care technology. The smart ward initiative will include technologies such as continuous vital sign tracking with an integrated dashboard for real-time patient monitoring, a unified communication system for improved team collaboration, inpatient teleconferencing systems for remote consultations and patient education, chatbot agents for patient education, and the integration of AI analytics into clinical systems. The initiative also envisions a centralized virtual workforce that remotely supports inpatient care and ground staff.

To achieve the smart ward vision, substantial changes in staff behaviors and workflows are required**.** Thus, the successful implementation of technology depends not only on the effectiveness of the technology itself but also on how well an organization and its staff can adapt to these new systems—a concept known as “organisational readiness for change” [8,9]. In the context of digital transformation, such as smart wards, organizational readiness reflects the extent to which staff and systems are collectively prepared—psychologically and behaviorally—to adopt and sustain technological innovations. According to Weiner [9]*,* two key constructs underlie readiness: (1) change efficacy (the shared belief in the organization’s capability to execute the change successfully) and (2) change commitment (the collective resolve to implement the change). Multiple factors may influence these constructs, including leadership support, local policy, clear timeframes, resource availability, staff training, and a culture for safe experimentation and learning (ie, sandbox environments) [9-11]. Together, they determine the degree of preparedness in an organization, both psychologically and behaviorally, to implement change [9]. When readiness is high, the implementation of a proposed change is effective, while low organizational readiness leads to resistance to and avoidance of change [9,12-14].

Although several implementation and organizational change frameworks exist; such as the Consolidated Framework for Implementation Research, and Kotter’s change model, Weiner’s organizational readiness for change model was chosen for its specific focus on the psychological and collective capability to implement change Unlike frameworks that emphasize structural, procedural, or leadership-driven dimensions, this model centers on the shared commitment and confidence of staff to enact change. Through examining how staff perceive readiness for the smart ward and the organizational conditions that support it, the study refines the understanding of how change commitment and change efficacy manifest in high-technology, team-based clinical contexts. Although there is an increasing global interest in smart hospital initiatives, most existing research focuses on the technological infrastructure, digital capabilities, or outcomes of smart technologies [15]. Far less is known about the behavioral and contextual factors that shape successful adoption in a smart ward context. Addressing this gap, this study examines the enablers and barriers to organizational readiness for change as Alexandra Hospital embarks on its smart ward initiative, using the framework proposed by Weiner as the analytical lens.

## Methods

We conducted a qualitative study to explore health care staff members’ readiness for the smart ward initiative. The project was run during an initial pilot testing phase, in which some, but not all, technologies had been deployed. This study is reported according to the Consolidated Criteria for Reporting Qualitative Research (COREQ) [16].

### Ethical Considerations

The study was reviewed and approved by the National University of Singapore Institutional Review Board for Social, Behavioral, and Educational Research studies (NUS-IRB-2023-327). All participants provided written consent to take part in the study. It was emphasized that the study was voluntary and participants could withdraw at any time. Participants received no compensation for taking part in the study. Data were deidentified and participants were assigned a unique ID number.

### Setting

**Figure 1:**
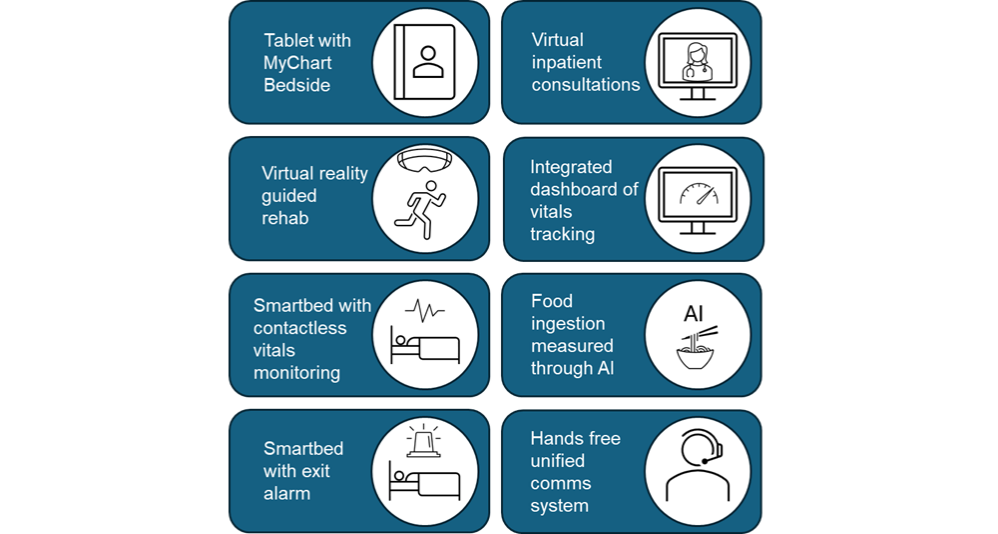
Summary of the planned smart ward technologies. AI: artificial intelligence.

Alexandra Hospital, part of the National University Health System, provides care across the continuum, from acute to subacute rehabilitative services. The smart ward initiative, based at Alexandra Hospital, incorporates a suite of technologies (Figure 1). These technologies enhance access to personal health information for patients (via the MyChart Bedside) and clinical data for providers through smart bed systems for continuous vital sign monitoring, automatic bed exit alarms, computer vision, and AI-calculated food consumption, and an integrated dashboard for data analysis and visualization. The initiative also introduces new modes of care, such as virtual reality-guided rehabilitation and virtual inpatient consultations, alongside improved communication through a unified communications system. At the time of this study, some but not all of the technologies had been deployed in the ward.

### Participants

We used a purposive sampling approach to capture the views of different departments involved in smart ward development or staff working within the inpatient setting, where the initiative is being implemented. Our inclusion criteria were as follows: staff employed at Alexandra Hospital during the study period; individuals involved in smart ward development or clinicians working within the smart ward; representatives from nursing, medical, IT, operations, or health care redesign departments; the ability to provide informed consent; and the ability to participate in an English-language interview. Exclusion criteria were staff not involved in or exposed to the smart ward initiative (ie, external vendors, temporary or locum staff) and the inability to provide informed consent. The choice of representatives was made as these departments were collectively involved in the design or implementation planning for the smart ward, or represented the main clinical groups affected by the smart ward transformation.

Participants were contacted by email or phone messaging. A research team member (with no direct relationship with the participant) followed up with the potential participant to discuss the project, obtain written informed consent, and arrange a convenient time for the interview. Recruitment continued until data saturation had been reached, defined as the point at which no new codes, themes, or perspectives emerged from successive interviews, and the research team agreed that additional data would not add further conceptual insights. Saturation was assessed iteratively during data collection among the study team. No participants declined to take part, and no participants received compensation to take part in this study.

### Materials

The interview guide was informed by the organizational theory of Weiner’s implementation effectiveness framework by [9], which is widely used to assess organizational readiness for change in health care settings [17]. This framework was selected because it provides a structured lens to examine the psychological (eg, change commitment) and structural (eg, change efficacy) factors that influence an organization’s preparedness to implement change. This makes it particularly suitable for examining health care workers’ perceptions before implementing a complex intervention, such as a smart ward. The interview topics included participants’ general views, awareness, and understanding of the smart ward initiative; perceived benefits and challenges; and preparedness, including available resources, managerial support, required skills, and value alignment. JS drafted an initial guide and subsequently shared it with the broader project team for feedback. The interview guide was also developed iteratively as new topics emerged during the interviews to ensure responsiveness to participants’ experiences. For instance, although we originally asked about the impact of the smart ward on staff-to-patient interactions, many participants also reflected on staff-to-staff interactions. Some also raised questions regarding the suitability of certain patients for the smart ward environment. These topics were subsequently incorporated into later interviews to systematically explore this emerging dimension. Despite these additions, all interviews continued to cover the same foundational domains related to organizational readiness, ensuring comparability across participants.

### Procedure

Interviews were conducted in pairs by 4 female health services researchers trained in qualitative methods (JS, HWL, CK, and EHHC) from August 2023 to February 2024. Interviews were conducted using a semistructured interview guide, were audio recorded, and transcribed verbatim. Interviews were conducted in a quiet place that was convenient and acceptable to the participant. On average, the interviews lasted 47 minutes (range 29-69 min).

### Data Analysis

We used a framework analysis approach, applying a hybrid deductive-inductive approach [18]. A deductive structure was based on the theory of the organizational readiness for change framework proposed by Weiner, which guided the initial coding. Inductive coding captured emergent insights in each domain. To contextualize the framework to our study, the project team reviewed the original definitions of the framework domains: change efficacy, change commitment, and contextual factors. The team then contextualized these definitions to our study through discussion, drafting a preliminary codebook. Three researchers (JS, HWL, and AA) then independently coded a single transcript sentence by sentence using the draft codebook and met to discuss coding assignments and clarify definitions. This exercise was repeated on a second transcript to resolve any remaining questions. All subsequent transcripts were coded by a single coder (HWL or AA) and checked by a second coder (JS) to ensure consistency. Differences in opinions were mutually reconciled through discussions among the team. Codes were subsequently grouped into subthemes within the framework domains through team discussions, allowing new concepts to emerge while retaining theoretical alignment. All coding and analyses were conducted in Microsoft Word.

## Results

We interviewed 19 participants from different professional backgrounds, including clinicians and support staff (Table 1).

**Table 1:** Participant characteristics (N=19).

Job function	Participants, n (%)
Operations	1 (5)
IT	3 (16)
Nursing	8 (42)
Physician	5 (26)
Health care redesign manager	2 (11)

**Figure 2:**
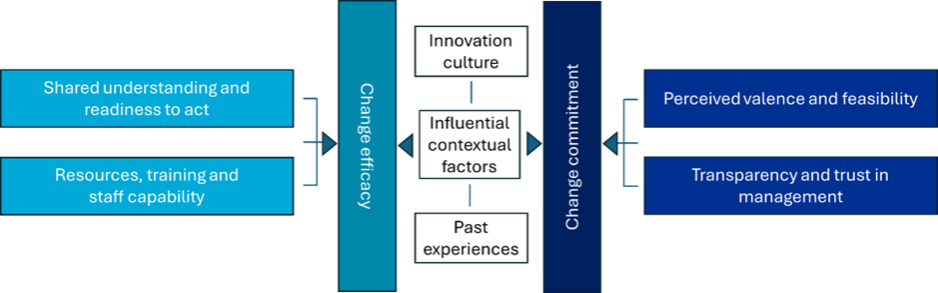
Mapping of inductively derived subthemes to the 3 key domains in the organizational readiness for change framework proposed by Weiner [9].

Results are organized according to the 3 key domains of the theory of organizational readiness for change proposed by Weiner (change efficacy, change commitment, and contextual factors), and 6 subthemes under these domains (Figure 2). We identified several enablers and barriers impacting the smart ward initiative (Table 2). A broader description of the influencing factors is provided in the following section. Additional supporting quotes are included in Multimedia Appendix 1.

**Table 2:** Summary of key enablers and barriers by organizational readiness domains (change efficacy, change commitment, and contextual factors) and the 6 subthemes.

			Enabler		Barrier
**Change commitment**					
	Perceived valence and feasibility	Value perceived Alignment on value across departments Perceived as appropriate for general medicine wards		Slow roll-out Limited integration Failure to meet expected needs Overreliance on technology Reliability questioned Alarm fatigue or desensitization	
	Transparency and trust in management	Trust in management Active stakeholder engagement Minimal expectations for high involvement		Minimal ground-nurse engagement Fear of failure or perception that innovation is not a priority Cultural norms impacting openness	
**Change efficacy**					
	Shared understanding and readiness to act	Good awareness of the initiative Facilitation of interactions between teams		Inconsistent communication Misalignments on purpose	
	Resources, training, and staff capability	Ground-up training initiatives Troubleshooting support		Delayed training or unclear responsibility for training Insufficient staff or time to engage with new technology Need for external funding	
**Contextual factors**					
	Innovation culture	Sandbox culture Keeping up to date with technology developments Raising staff awareness of technology		Complexity of the “sandbox” approach in the clinical setting	
	Past experiences	Prior positive experience with technology		Prior negative experience with technology	

### Change Commitment: Perceived Valence and Feasibility

Participants broadly agreed that the smart ward initiative held value and had potential to improve efficiency, reduce overtime, and allow more dedicated time for direct patient care. Their comments reflected enthusiasm and a shared sense that the change was desirable:

It’s to help us, to make our job easy, to elevate, to help to reduce the workloads.

Nurse ID 04

Yeah, for sure. I will love to work in an environment like that. Everything is so streamlined. Yeah, that would be good.

Physician ID 12

However, during the pilot phase, expectations were often unmet. Many expressed frustrations with implementation delays, lack of system integration, and a failure to reduce workloads. Others shared that pilot testing had resulted in “double work,” with old and new processes carried out simultaneously because the new process was not trusted. Health care redesign staff echoed these concerns, noting early resistance and uncertainty around pilot technology:

I think so long already until [smart ward], I think they need to expedite.

Nurse ID 05

Because we don’t totally trust it right? Because it’s totally new and it hasn’t been totally tested out. So you want to do things the old way as well.

Physician ID 12

When we tried to implement anything new, there’s always resistance. Because I guess it’s, at first, it’s just umm, they didn’t make sense out of it. It’s like. At first, it’s not going to be everything smooth. It’s going to be a lot more work at the beginning for them. So it’s normal that they may feel hesitant about it.

HCRD ID 18

Concerns about overreliance on technology, alarm fatigue, and erosion of human interactions were also common:

Yeah, definitely. There are some things that require our own judgment. I mean when we are trained, we need to correlate that to how the patient is clinically, like what are the symptoms, how they look, how they appear, what are some of the signs that they have.

Physician ID 14

I think it’s helpful but I also don’t know like how reliable it will be, because like. for example, patient is just agitated for a while, then there’s a spike in the heart rate. Then umm, if we’re going send alerts each time, then it’s not going to be helpful.

Physician ID 16

OK, since I can already get my vitals there, right, so it might decrease my bed time interaction.

Nurse ID 06

From a systems perspective, IT and operations staff highlighted the need for reliable infrastructure and clear governance to prevent failures and excessive maintenance burdens. Health care redesign staff added that bureaucratic barriers contributed to delays and hindered innovation:

While it is important to have all this checks in place [security checks prior to implementation], I’m not too sure that they understand that sometimes it’s just a pilot.

HCRD ID 18

Imagine today there’s a concept of virtual nursing where one nurse can take after 50 patients over screens.... But if the technology fails, what happens to that 50 patients?

IT ID 08

### Change Commitment: Transparency and Trust in Management

Perceptions of strategic involvement in the smart ward initiative varied across professional groups. Some ground nurses felt excluded from the design and decision-making process despite being the primary users, contributing to a sense of disconnection. In contrast, physicians were less concerned about their level of involvement, noting that they lacked time to innovate but were open to providing discrete feedback. IT and operations staff reported a more structured advisory role—focused on implementation rather than shaping the initiative’s direction:

So we need to know everything that is going on in the ward, but when they didn’t inform us, we feel like we are not a part of it. So yeah, that’s how we feel.

Nurse ID 04

To come up with those ideas and the vision and the direction, I don’t think most of us honestly have the time.

Physician ID 13

No, I think we do have quite regular updates and meetings to keep each other informed.

Operations ID 01

IT and health care redesign staff generally viewed management as supportive of innovation but recognized that maintaining momentum required constant buy-in. Others felt that innovation was not always a top priority, and that fear of failure among senior management constrained their ambition. Nurses were the most skeptical, describing trust as contingent on seeing concrete results from technological investments:

I think sometimes it’s the fear to fail that actually hinders the progress.

HCRD ID 19

We have a very strong management that says, no matter what, we will be behind support you, you will get this. You think that other places can have this?

IT ID 02

### Change Efficacy: Awareness and Shared Understanding

Nurses and physicians valued the initiative’s aims but felt uncertain about next steps and timelines, reflecting limited communication and shared understanding. In contrast, IT and redesign staff, who were closely involved in planning, described the project as a concrete realization of digital transformation goals:

What is happening now? Are we on to this phase? What phase now? What are we waiting for now? when are the trainings going to be?

Nurse ID 05

Smart Ward is a place that where we can put our dreams into real life.

IT ID 02

Health care redesign staff noted that inconsistent communication and technical jargon often hindered cross-departmental engagement, although they actively worked with IT to bridge these gaps. They described ongoing efforts to involve all stakeholders but acknowledged that achieving full transparency remained difficult:

You know, sometimes we try to like, you know where we have like new products that we want to bring in, new technology that we want to bring in, we will try to invite everyone.

HCRD ID 19

Actually I think IT and the business right can never have a common language because our language is so much more technical right? We can only start by teaching them. So when they time come to us with the business requirements, we translate that into a technical term and then we share, this is what you call it.

IT ID 08

### Change Efficacy: Resources, Training, and Staff Capability

All participants recognized the need for adequate resources for technology implementation. Nurses highlighted that insufficient staffing would hinder technology use, while IT struggled to hire and retain skilled staff due to competition with industry roles. Health care redesign staff also called for greater IT resourcing:

There are certain personnel in IT that we work with very closely. But if you were to ask me if they are dedicated just solely doing our projects, I don’t think so.

HCRD ID 19

It’s always difficult because being in public healthcare, right, our pay is relatively lower than private sector. So, you always have this fight with the private sectors that you want somebody good, right? Yeah, but you cannot pay.

IT ID 08

IT staff further speculated on the long-term scalability of smart ward, given budget constraints and ongoing operational costs. Others noted the challenge of needing external funding to support their efforts:

We are running short of money for public healthcare. Because over the years we have been commissioning more and more IT systems, right and each time we commission an IT system, it actually draw an operating cost. It increases our operating cost. And if we don’t start to consolidate them, some of the systems, right, this will just continue to grow.

IT ID 08

We ourselves, we are also supposed to look for the funds, for our pilot projects.

HCRD ID 19

All teams emphasized the importance of training to ensure competency on the ground but described inconsistent provision and unclear responsibility for ongoing training. This lack of clarity and execution led to a poor understanding of pilot technology and inconsistent user compliance across departments. Health care redesign staff were unconcerned about in-depth training, citing cross-functional support. IT viewed ongoing training as the responsibility of system owners rather than IT itself. Consequently, nursing often initiated in-house training programs, but this did not extend to other departments, creating knowledge gaps across functions:

So definitely the team needs to be equipped with skills, like understanding the tech landscape right, the jargon, and basically the landscape, because as an administrator when we have all this data.

Operations ID 01

Because sometimes the training will come only if the thing is there already.

Nurse ID 05

Usually we will get the vendor to provide the training to the users before they hand over the system for users to maintain it. So that will be their [system owners] responsibility to share the materials with the staff.

IT ID 15

### Contextual Factors

Participants generally viewed Alexandra Hospital as a “sandbox” for trials and innovation, which fostered a belief in the feasibility of the smart ward. However, they also recognized that such experimentation is more complex in a hospital environment than in other industries. Several participants emphasized the role of prior technology exposure and mindset in shaping readiness. Those with positive past experiences were more open to change, whereas those with past system failures were more cautious. Strategies such as routine “tech scans” to stay informed and efforts to update staff on technology advancements to boost engagement were seen as beneficial, particularly to encourage those who are comfortable with the status quo:

We are designated sandbox [ie, a site for experimentation and testing of innovative ideas] from MOH, okay. We can do, try, trial, and we are not afraid of failing.

IT ID 02

So I have had to deal with system breakdowns. The backup system was extremely difficult to use for everyone. So it definitely took a toll on everybody.

Physician ID 12

### Cross-Cutting Influences on Collective Readiness

Differences in staff perceptions reflected not only professional roles but also the hospital’s culture. Frontline clinicians, who had less decision-making authority, mainly described the smart ward regarding its day-to-day effects on workload, communication, and patient care. In contrast, staff in IT, operations, and health care redesign, who had greater strategic oversight, tended to focus on systems change factors, including integration, scalability, and long-term feasibility. The hospital’s culture of cautious innovation, which encouraged experimentation but was often limited by bureaucratic processes or a fear of failure, also shaped readiness. Taken together, collective readiness for change was shaped not only by perceptions of the technology’s value but also by how authority and organizational culture shaped staff experiences and their sense of ownership in the change process.

## Discussion

### Principal Findings

As technology continues to be deployed in clinical environments, assessing the factors influencing change readiness is essential for its successful implementation and sustained use. We conducted a qualitative study to gauge the organization’s readiness to change for the smart ward initiative at Alexandra Hospital. We interviewed a diverse cross-section of health care providers, operational support staff, and the in-house innovation team to identify facilitators and barriers. We found that participants generally saw value in the concept and trusted management. Many appreciated active engagement in the initiative and viewed the hospital’s sandbox culture as a key enabler of innovation. However, challenges were evident, including unclear implementation timelines, inconsistent training, and limited resourcing for innovation. Concerns about the overreliance on technology were also prominent, with staff wary of its impact on clinical judgment and system reliability.

A key aspect of change commitment (a core construct of the organizational readiness for change framework by Weiner) is the perception that a proposed change holds value, which is essential for successful implementation [9]. This aligns with the 8-step change model developed by Kotter and the diffusion of innovation theory by Rogers, which emphasizes that if clear benefits are unrecognized, it can lead to a lack of urgency, reduced participation in the process, and may ultimately stall the implementation process [19,20]. In our study, we found that stakeholders from different departments collectively saw the initiative as beneficial, but the underlying perspectives on purpose differed between teams. Similar challenges have been observed in other digital transformation contexts, where divergent stakeholder views have led to misalignment and ultimately constrained implementation efforts [21].

Another driver of change commitment is engagement with stakeholders. Effective and early communication with stakeholders can help build buy-in, address concerns, align expectations, and maintain engagement. In contrast, poor engagement often leads to suboptimal adoption. For example, hospitals that introduced electronic medical records without consulting staff often faced resistance [22]. Conversely, those that involved staff early in technology design and roll-out found that implementation was facilitated [23,24]. Our findings indicated that participants had varying degrees of engagement. Participants closely involved in the initiative viewed the process as collaborative, while some frontline staff felt less informed. To improve engagement, the nonadoption, abandonment, scale-up, spread, and sustainability framework recommends structured, transparent, and multidirectional communication to engage stakeholders and facilitate adoption [25]. Within the framework proposed by Weiner, such communication can enhance collective change commitment and efficacy by fostering a shared understanding, confidence, and ownership of the change process. Interventions could include workshops, multiple communication channels, tailored messaging, and continuous feedback loops to enhance stakeholder commitment.

Of the contextual factors influencing readiness for change, participants shared that having an innovation culture at Alexandra Hospital is a key enabler for the smart ward initiative. Although relatively new in health care, the “sandbox approach” provides a low-risk environment for testing new ideas or technologies without full implementation [26,27]. This approach may be beneficial when introducing disruptive technology, such as that in the smart ward initiative, as it provides space for learning and experimentation without full implementation [26,27]. The sandbox approach aligns with the model by Weiner, emphasizing that perceived capability (which can be acquired through experimentation) is a core component of change efficacy [9]. However, although there was a belief that a sandbox culture exists at the hospital, the infrastructure to support it was lacking. Clear communication on the purpose and objectives of the sandbox approach could help to establish the support required to achieve a true sandbox environment [28].

Training is another factor in capability development. We found that delayed or inconsistent access to training and a lack of clarity on who is responsible for training led to knowledge gaps on the ground. A lack of training may become problematic, as technology literacy is a known barrier to technology adoption and sustainability [29]. According to the organisational readiness for change model proposed by Weiner, ensuring that stakeholders feel competent and supported is essential for successful implementation, a point also mirrored in the Consolidated Framework for Implementation Research [30]. Effective training overcomes resistance to change by improving awareness, competence, and confidence [20]. These findings align with prior studies showing that insufficient or poorly timed training undermines readiness, while targeted, context-specific programs enhance staff confidence and sustain adoption [25,31]. Efforts should focus on providing timely access to training, defining responsibilities for training delivery, and tailoring content to different stakeholder groups to accommodate varying levels of digital literacy and learning needs [32].

Our findings have several practical implications for hospitals planning or implementing smart ward initiatives. We suggest several practical strategies to support the successful implementation of similar smart ward initiatives. First, efforts should focus on building awareness of the value of such initiatives to ensure a buy-in and consistent understanding. Second, implementation strategies should actively engage staff across all levels, not only clinical leads or innovation champions, to ensure that diverse perspectives are captured and foster collective ownership of change. Third, to operationalize the “sandbox” culture that participants value, institutions should establish concrete support mechanisms, such as a streamlined procurement mechanism, expedited technology safety reviews, and iterative feedback loops to rapidly trial and refine technologies in real-world settings. Fourth, training frameworks should be developed early, with clearly assigned responsibilities for delivery, content tailored to varying digital competencies, and adequate protected time for staff to learn. Finally, leaders should visibly support implementation efforts, actively monitor progress, adapt as needed, and establish mechanisms to surface and address frontline concerns as systems evolve. Together, these actions can improve organizational readiness and enable more effective, inclusive, and sustainable digital transformation in hospital settings.

### Strengths and Limitations

We conducted a qualitative study that provides in-depth insights into the readiness of clinicians and support staff members for change. We used an established framework to explore organizational factors influencing technology adoption, which is a clear strength. Our qualitative approach also allowed us to identify context-specific enablers and barriers that may be overlooked in a survey approach. However, the generalizability of our results may be limited, as our data are from a single institution. Although we sought to capture a range of perspectives, the representation from some departments (notably IT and operations) was smaller. In these cases, we included senior management who were directly involved in system-level planning, reflecting the primary function of these departments at this stage of the project. For frontline staff, we recruited a broader sample to capture the experiences and views of those impacted by the smart ward. Although we acknowledge the underrepresentation of some professional groups, thematic saturation was achieved across the main domains of readiness, and the inclusion of both strategic and frontline viewpoints enabled us to interpret the findings holistically. We also cannot rule out sampling bias and the risk of social desirability bias in participant responses. We attempted to mitigate the latter by emphasizing participant anonymity. Another limitation of this study is that we used the organisational readiness for change framework by Weiner to inform the development of the interview guide and to structure the initial deductive analysis. This dual use may have reinforced existing theoretical constructs and constrained the emergence of entirely novel themes. To minimize this risk, we incorporated inductive coding within each domain to capture insights that extend beyond the original framework. Finally, as with all qualitative research, the findings reflect the interpretations of the research team, although efforts were made to enhance rigor through team coding.

### Conclusions

By identifying and addressing pertinent factors that influence readiness for change, the enablement of technology implementation is possible. Efforts should focus on project awareness, particularly among ground staff, the development of training frameworks, and the adequate prioritization of an innovation culture. Furthermore, alleviating commonly reported technology concerns, such as overreliance, loss of human touch, and reliability issues, will be key to adoption and sustainability.

## Data Availability

All data generated or analyzed during this study are included in this published article.

## Appendix

Multimedia Appendix 1 Additional supporting quotes organized by subthemes.

## Abbreviations

AI: artificial intelligence
