# Supplementary material for: Organizational Readiness for Change in the Era of Smart Hospital Wards: Qualitative Study of Health Care Workers’ Insights
Source: JMIR Hum Factors. 2025 Dec 18;12:e81932. doi: 10.2196/81932 (PMC12757706; doi:10.2196/81932)
Supplement: Multimedia Appendix 1 [file humanfactors_v12i1e81932_app1.pdf]

Organisational Readiness for Change in the Era of Smart Hospital Wards:
A Qualitative study of Healthcare Workers' Insights

Supplement 1: Additional supporting quotes, organised by sub-themes

**Sub-theme: Perceived valence and feasibility**
Nurse ID05- "I'm hoping that's it's something that really trying to help with the current manpower, with the manpower crunch, we don't have enough manpower."<br>*Nurse ID03- "The main thing, patient safety. Out of all these changes, as compared to other wards, definitely the smart bed. There were instances where we managed to prevent fall. The heartbeat monitoring as well, it [smart bed] alarmed us accordingly, so we escalate the issues to the doctor's on time."*<br>*Nurse ID05- "I think they need to expedite [the smart ward]…the one that they promised, until now we don't have."*<br>*Nurse ID03- "Yeah not as I expected, because whatever I'm doing, it's the same."*<br>*Nurse ID07- "The newer batches of nurses coming up, they might be taught the use of technology more, less like manual assessment of heart rate. So I think that is a problem that will be coming in the future, where we have more young nurses coming in where they only know how to use the technology. What if the network is down or power outage."*<br>*Nurse ID09- "There are still things that they [technology] cannot perform compared to human beings, like nurses who has really the background knowledge and the experience on how to really take care of patients."*<br>*Doctor ID16- "Like in terms of nursing, to reduce the burden."*<br>*Doctor ID14- "So currently the nurses will go and manually take the vital signs and then there will be report back if there's any abnormalities. So it is quite labour intensive. If the bed can do that, then definitely it would would be good for the manpower. Take some load off the nurses."*<br>*HCRD ID19- "I mean, it would definitely be easy if they have some invested interest in the item that we are trying to bring in, then obviously things just move much faster. But if it is a technology that we try to implement and they don't see the value of it, then I would say it's definitely very difficult."*<br>*HCRD ID18- "It's going to be a lot more work at the beginning for them. So it's normal that they may feel hesitant about it. Like, is it worth my time doing it? Or testing it out?"*<br>*HCRD ID08- "Sometimes the solution is very good. But we have a lot of red tapes, like IT red tape."*<br>*IT ID15- "So obviously we have a lot of clearance stages required, to assess whether or not the system is secure enough to safeguard our confidential data. So, this one, typically will require three to six months for the assessment."*<br>*IT ID15- "I think the challenge is always to safeguard our patient info. So, it's kind of like placing a heavy compliance burden on IT team."*<br>*IT ID08- "Each time we commission an IT system, it increases our operating cost. And if we don't start to consolidate them, some of the systems, right, this will just continue to grow. It has already grown to a stage where IT operating expenditure is close to 70% of the entire IT budget. Meaning to say you are left with maybe 20% to spend on new projects."*<br>*Ops ID01- "I think there are two problems to address, first is the patient themselves, they can self-help. The other area would be the staff we kind of cut down the manual work, saving resources."*<br>*Ops ID01- "Where in a smart ward as a patient, maybe I don't even need to come into a hospital to even have that consult or care."*
**Sub-theme: Transparency & Trust in management**
Nurse ID03- "I think the engagement, is make their promises happen, not like their words only. Like to make it happen. So keep us updated, so things like that are more important. I mean because you keep the hype, you keep the excitement."<br>*Nurse ID05- "No, you [management] don't understand how we feel."*<br>*Nurse ID07 "Yeah, I do feel that I'm adequately supported."*<br>*Nurse ID07- "Lets say it's a function that I'm not sure of how to use. Usually I'll ask [Nursing seniors] and they are all very willing to share."*<br>*Nurse ID09- "(Interviewer: So generally, do you feel supported?) They listen. Because my chief nurse, yeah, this is the culture that she want us to really understand everyone… So we are heard."*<br>*Doctor ID13- "I mean, if let's say, they have like option one, option two, option three; which do they think would be the most feasible on the ground? that kind of thing I don't mind….But to come up with those ideas and the vision and the direction, I don't think most of us honestly have the time."*<br>*Doctor ID14- "I think I wouldn't mind. It's just that because we rotate through different hospitals I may not be able to experience it on my own, so I'm not sure whether that would be very relevant."*<br>*HCRD ID19- "When we have like new products that we want to bring in, new technology that we want to bring in, we will try to invite everyone."*

HCRD ID19- "So I don't know if they [management] really have reservations about innovation, maybe innovation is not the top of the list. Maybe providing sufficient bed space for patients is top of their priority. Our CEO has less constraint of having the fear to fail. I think sometimes it's the fear to fail that actually hinders the progress."<br>*IT ID15- "I mean generally the users will be the main people deciding this right?[technology investment] Because the money come from them. But we will give the advice accordingly.*<br>*IT ID02- "We have people that are very daring, a lot of ideas, not afraid of like failing it."*<br>*Ops ID01- "We need to execute this project with the healthcare redesign team. That is when we hear more details right about the design concept, what are the planning of all those behind things like this."*
**Sub-theme: Awareness & Shared understanding**
Nurse ID05- "They've been telling our reporting officers all the time. Yeah, I think since we started, we cannot see anything at all coming."<br>*Nurse ID03- "Is basically to simplify what nurses are doing so that we can we really spend time with our patients."*<br>*Nurse ID03- "I'm not too sure how this works out because everything is under the carpet at the moment. I have no idea. Yeah, we have no idea when they are going to implement it."*<br>*Nurse ID04- "I am not too sure if they tell our ward sisters. But when I came to work that day, I asked the person [from healthcare redesign], what you guys are doing here? Then she say oh we're going to install this this this. So that's when I knew it."*<br>*Doctor ID13- "I've seen the big bosses bring around people to marvel over it. Yeah, but that's what it is. I mean, I'd appreciate it if they are extending it to other wards, at least to know kind of what to look out for, some advanced warning would be good. Because I mean, in the middle of the night, if you are on call or you just started new adult ward, the last thing you want to do is 'where's everything is' kind of situation".*<br>*IT ID08- "I do not know who came up with it first right, rather it is more like a joint dream."*<br>*IT ID08- "We work very closely with this team, Healthcare Redesign Team. So basically umm the team always involve IT ...to have a discussion with the vendors to understand about technologies and also to find a way to achieve the common goals."*<br>*IT ID08- "Yeah, as compared to the conventional way, you need to raise paper, you get approval before you can do a project. And when you do a project, it's very much like a waterfall kind of methodology. You do the requirements, study, design, whatever, whatever. And by the end of the whole cycle, three years passed."*<br>*HCRD ID19- "Maybe people are just too busy to read emails I guess, maybe it's also because there are a lot of emails circulating around and people, I don't know, tend to overlook or everyone is overloaded with a lot of different projects."*<br>*HCRD ID18- "It's supposed to be like a playground for us to test new care models and evaluate it."*<br>*Ops ID01- "We pretty much like, like we know, what is the concept and then you can see it happening in phases like first we have to order infrastructure that is ready then you start seeing the different technologies and the equipment coming in and the final piece is the integration and again that is the most critical and the important piece to just pull all the pieces together yeah."*
**Sub-theme: Resources & Staff capability**
Nurse ID05- "The thing is there and then we do training and then we are using it already and we don't even know the function. It is better that you go for the training before you use the item."<br>*Nurse ID06- "And I think because when we just started most of the PT and OT, I would say that they are not really aware on how to use the beds and the first stage of opening the smart wards, because I would say because they don't really receive the trainings. So we are the one that teach them what to use, how to use."*<br>*Nurse ID03- "I think the most important thing would be open mindedness….We can have training sessions, definitely we can train the nurses but if the nurses don't have mindset, it's not going to work. That's what I feel."*<br>*Doctor ID12- "(Interviewer: OK, so training then, it's critical.) That's right. Because, I mean, it's great to give us an e-learning module but by the end of the 30 minutes of watching multiple videos I can't remember."*<br>*Doctor ID17- "I guess it depends if we're given appropriate training and things righ, and obviously it takes time to get used to things as well."*<br>*HCRD ID18- "I think we don't need to go in depth because if I go in depth, then I might as well take over their role right? (Laughing). Yeah, I think like just sufficient for us to know the basics."*<br>*HCRD ID19- "Probably like providing more beds for patients to make sure that they are able to be cared for, uh, almost as fast as possible is probably top on their list. That's why innovation maybe it's probably second or third."*<br>*HCRD ID19- "All of us is quite tied up because we are writing papers to different people trying to obtain money. So a lot of our attention gets like, divided out quite thinly actually."*<br>*IT ID08- "So you need people that are well verse in, let's say, the AWS cloud or the Microsoft Azure cloud. These are different skill sets from conventional projects."*<br>*IT ID15- "Generally, everyone have their own skill set, right? They are good in clinical stuff. Perhaps, IT ah, are supposed to be good in umm technology stuff. So I guess it will be good for users to know more about the usage of the system to fully utilize the features of the system itself. Yeah, usually we will get the vendor to provide the training to the ah users before they hand over err the system for users to maintain it. Yeah, usually would be the system owner's responsibility."*

IT ID08- "It happens in all kinds of projects, not just about Smart Wards, it's all kind of projects. Yeah, money is the first road block, we always have the issue with insufficient fund."<br>*Ops ID01- "I mean, I myself am very new to this as well. Yeah, but as we go along and then we start to learn."*<br>*Ops ID01- "The people on the ground like the doctors, like your nurses and even your housekeeping people, How to work on that bed? How to operate? Because there's suddenly so many buttons, like before your normal weight is maybe just a lever and another button somewhere."*
**Sub-theme: Innovation culture**
Nurse ID03- "The willing to change because we are actually dealing with a lot of like incoming nurses who are younger and younger. So I think we are more and more exposed to technology already."<br>*IT ID02- "We have a Smart Ward, we have places that we can playground, play with it and we have been given authority to sandbox it. We have a strong team to support it."*<br>*HCRD ID18- "So I think it's just, probably that culture in the urgent care centre they're generally more willing to try new things."*<br>*HCRD ID18- "Smart Ward would be the place that the nurses or doctors are free to try on like new Care Models. So it's called a Sandbox that err playground for, for us to try things."*<br>*HCRD ID18- "Well the smart ward is supposed to be able to kind of mimic that [sandbox approach], but actually if you were to ask me. Uh, it's not the 100 percent true space that we can just plug in and play."*
**Sub-theme: Past experiences**
Nurse ID03- "I think the interest about nursing informatics came from when I first went to the states for attachment back in 2018. Then I see how epic was so convenient, helping the nurses there to do their documentation."<br>*Nurse ID07- "Yeah, when I was a student, I've really started using technology quite a bit. So previously you know nurses have to manually check medications but with the help of the electronic medical records with the barcode scanners, it does reduce medical error to a certain extent."*<br>*Nurse ID10- "So before I worked in Philippines actually, as you know we are not really that techie yet. So at first I feel overwhelmed with the technology they have [Alexandra Hospital, Singapore] but after that I slowly get used to it."*<br>*Doctor ID 12- "So I'm lucky enough to have gone through different institutions, so I've had to learn different system. Even EPIC was new to me until this year. I wouldn't say I was prepared as in I had expected that change. And so I just had to learn to get used to it."*<br>*HCRD ID19- "Previously in the finance industry, when I was working with them, there were a lot of technologies that they were trying out. But I felt that at that point in time, why they were able to move so fast is that they were actually given a safe space to work without having red tape issues."*<br>*Ops ID01- "So using epic as an example. Like, we do have lots of support on it, like cluster level and of course training is definitely there."*
